# Combining ontologies and workflows to design formal protocols for biological laboratories

**DOI:** 10.1186/1759-4499-2-3

**Published:** 2010-04-23

**Authors:** Alessandro Maccagnan, Mauro Riva, Erika Feltrin, Barbara Simionati, Tullio Vardanega, Giorgio Valle, Nicola Cannata

**Affiliations:** 1 CRIBI Biotechnology Centre, University of Padua, viale G. Colombo 3, 35121 Padova, Italy; 2 Department of Pure and Applied Mathematics, University of Padua, via Trieste 63, 35121 Padova, Italy; 3 BMR Genomics, Via Redipuglia 21/a, 35131 Padova, Italy; 4 Department of Mathematics and Computer Science, University of Camerino, Via Madonna delle Carceri 9, 62032 Camerino, Italy

## Abstract

**Background:**

Laboratory protocols in life sciences tend to be written in natural language, with negative consequences on repeatability, distribution and automation of scientific experiments. Formalization of knowledge is becoming popular in science. In the case of laboratory protocols two levels of formalization are needed: one for the entities and individuals operations involved in protocols and another one for the procedures, which can be manually or automatically executed. This study aims to combine ontologies and workflows for protocol formalization.

**Results:**

A laboratory domain specific ontology and the COW (Combining Ontologies with Workflows) software tool were developed to formalize workflows built on ontologies. A method was specifically set up to support the design of structured protocols for biological laboratory experiments. The workflows were enhanced with ontological concepts taken from the developed domain specific ontology.

The experimental protocols represented as workflows are saved in two linked files using two standard interchange languages (i.e. XPDL for workflows and OWL for ontologies). A distribution package of COW including installation procedure, ontology and workflow examples, is freely available from http://www.bmr-genomics.it/farm/cow.

**Conclusions:**

Using COW, a laboratory protocol may be directly defined by wet-lab scientists without writing code, which will keep the resulting protocol's specifications clear and easy to read and maintain.

## Background

High-throughput technology has contributed to the large-scale studies on the characterization of populations of biological entities [1]. A variety of "-omics" disciplines, such as genomics [2], transcriptomics [3], proteomics [4] and metabolomics [5,6], have begun to emerge, with their own sets of instruments, techniques, reagents and software. The characterization of the "-omes" produces huge amount of data that would be impossible to process without Information Technology. The work of life scientists is also rapidly changing. Now a researcher deals not only with laboratory equipment and *in vitro *experiments but also with software and web resources, i.e. *in silico *experiments. Scientific protocols include a very broad spectrum of activities (whether manual or automated) to be executed at the work bench and/or on the computer. Computers play a central role in data production, collection, storage, hypothesis formation and experimentation [7]. Several sectors of science are becoming largely automated [8] and this aspect has been highlighted by the emergence of "e-Science" [9]. However, to reap the benefits of computers and consequently of automation, it is essential that scientists change the way in which scientific knowledge is described, reported and finally stored. In fact, two of the problems in contemporary life science research are the interpretation and the reproducibility of published experimental results. Hence there is urgent need for a formal representation of scientific knowledge, including procedures (e.g., laboratory protocols, bioinformatic workflows).

Laboratory protocols and experimental methodologies are indeed an integral part of research in life sciences. The way in which protocols are described is decisive in permitting the reproducibility and the successful replication of experiments. Normally, the detailed notes about the kind of experimental procedures and their order, the type of materials and the variety of methods used by a researcher are available only inside his research group or department. The information is then disseminated through the research community by scientific publications and as a consequence it becomes available for the use of scientists who are new to that topic. Every individual study rests on ad-hoc laboratory protocols, these are usually included in a "Materials and Methods" which are defined only in natural languages. This way of describing laboratory processes has many limitations for their repeatability, distribution and more importantly automation. This can lead to ambiguous statements and to vastly arbitrary interpretations. Textual representation is the best choice for readability but it does not promote the re-use of parts of the protocol description and does not give a global, structured vision of the whole process as well as not highlighting the numerous resources necessary for the execution of the experiment.

A researcher can spend weeks or even months to learn, set up, and apply new experimental techniques or protocols. Thus, a significant amount of time in the laboratory is spent learning techniques and procedures mainly published by other research groups. This is a never ending process for experimental life scientists since methodologies and their respective protocols are evolving at a dramatic pace. Moreover laboratory automation is becoming increasingly crucial in many fields of experimental research. In fact, many wet-lab activities are becoming dependent on laboratory robots [10]. Bioinformatics encompasses automation in all the aspects related to biological data, including data collection, management and analysis. Two levels of formalization are required: one for the entities and operations deployed in protocols and another for the protocols themselves that can combine manually executed and automated procedures.

Ontology is one of the strategies for the structured and formalized representation of a chosen knowledge domain domain in a formal way, helping to remove ambiguity and redundancy, detecting errors and allowing automated reasoning. Ontologies describe the entities of the specific domain but do not specify how these entities should be used and combined.

Workflows can do this job. A workflow is a representation of a sequence of operations, declared as the work of a person, a group of persons, or machines. Workflows permit the description and the orchestration of complex processes in a visual form, capturing human-to-machine interactions within those processes. Several disciplines adopt workflows systems for the automation of data processing through a series of processing stages.

In this paper we propose a method for the formal representation of biological laboratory protocols that combines the unambiguous semantic of ontologies with the expressive power of workflows. Based on this approach, we have developed COW (Combining Ontology and Workflow), an add-on for the workflow editor JPEd [11] to design laboratory protocols, that integrates both ontologies and workflows. The software allows designers of protocol-workflows to select concepts from a domain specific ontology and to include them in their workflows.

### Laboratory protocols

Several on-line resources are available for retrieving information about life-science protocols and experiments. Since 1997, the Science Advisory Board (SAB) [12] has been working to improve communications between biomedical scientists and suppliers of laboratory products and services. SAB also maintain an extensive database of protocols divided by techniques.

Protocol-Online [13] appeared in 1999 on the web as a database resource for research protocols in a variety of life science fields such as cell biology, molecular biology, developmental biology, and immunology.

In 2004, the Nature Publishing Group (NPG) launched Nature Methods [14], a monthly research journal on novel methods and significant improvements to laboratory techniques in the life sciences and related areas of chemistry. In addition, Nature Methods includes a Protocols section describing established methods written using 'bench terms'.

In 2006, JoVE [15] started to publish on-line video-protocols. The user is not required to read through a written protocol but can simply watch a video. Each video article includes step-by-step instructions for an experiment, a demonstration of equipment and reagents, and a brief discussion, with experts describing possible technical problems and modifications [16].

In the same year, Nature Protocols [17], became available as a cutting-edge on-line journal for biological and biomedical protocols. Protocols, written in natural language, are organized into logical categories in order to be easily accessible to researchers. They are presented in a 'recipe' style providing step-by-step descriptions of procedures that users can take to the lab bench and immediately apply to their own research.

As an example of a protocol for *in silico *experiments, Huang et al. [18] describe how to use the DAVID bioinformatic resources for the analysis of large gene lists derived from high-throughput genomic experiments, including how DAVID modules are able to help users to extract biological meaning from the given gene list and how individual modules should be used either independently or jointly. The reader can find the procedure easier to follow to reproduce the study.

The approach used for describing a computational procedure is also adopted for laboratory protocols. For instance, the protocol suggested by Fiegler [19] is organized into several sections; first, a list of materials used in the experiment including equipment, materials and their set up is provided. The second section is a step-by-step description of the methodology used. Critical steps that must be performed in a very precise manner and all toxic or harmful chemicals are highlighted. These warnings are tagged by the heading

#### Critical step and Caution

Unlike the articles in the previously cited journals, in Nature Protocols the author is also asked to report the timing and possible troubleshooting in order to give an idea of the duration of the procedure and on how to troubleshoot the most likely problems. Writing protocols using the same pre-defined template will help to understand the procedure, as well as the critical steps and implementation of the technique reported in the published study.

In laboratory protocols there are numerous examples of ambiguous sentences. In fact statements that can be interpreted in different ways can introduce uncertainty as to how the procedure should be performed. For example the instruction "Remove the supernatant and dry the precipitated DNA briefly before washing with 100 *μ*l of 70% ethanol" introduces an ambiguity of the term "briefly", which may indicate different lengths of time. It could mean 30 seconds, 5 minutes, 10 minutes or a longer time. The term "gentle" in the instruction "Transfer slides into a solution of 0.1% sodium dodecyl sulphate and incubate for 5 min with gentle shaking." can be arbitrarily interpreted. This problem could be overcome by providing a single value or a range of admissible values, depending on the activity performed, which can help reduce the ambiguity in the meaning of the term.

Finally, the writing style of Nature Protocols is not intended to facilitate the automation of procedures. A computer machine will not be able to read it, interpret it and then replicate the original experiment.

### Ontologies

The need to unambiguously classify the huge amount of data available as well as precisely define their semantic relationship has increased the need for formal knowledge representation. In the 1980's, the ontologies entered the computer science field as a way to provide a simplified and well-defined description of a specific domain or an area of interest. An ontology defines "a set of representational primitives with which to model a domain of knowledge or discourse" [20]. Ontologies provide a common shared vocabulary to model a domain, defining the types of objects and concepts that exist with their properties and relationships. Ontology can be classified according to the subject of conceptualization into [21]:

1. general or common ontologies, defining concepts to represent common sense knowledge, reusable across domains;

2. top-level ontologies, defining very general concepts independent of a particular domain such as space, time, object, event, etc., and providing general notions from which all root terms in existing ontologies should be related;

3. domain ontologies, defining concepts within a specific domain and their relationships; the concepts in this type of ontology are usually the specialization of concepts already defined in a top-level ontology;

4. task ontologies, defining concepts related to the execution of a particular task or activity and providing a vocabulary of terms used to solve problems associated with task that may or may not belong to the same domain;

5. application ontologies, containing all the definitions needed to model the knowledge required for a particular application.

Recently, we have seen an explosion of interest in ontologies as models to represent human knowledge. Ontologies are now extensively used in applications related to areas such as knowledge management, natural language processing, e-commerce [22], web services [23], intelligent information integration, bioinformatics [24], education, life sciences [25] and medicine [26], and in widely adopted technologies such as the Semantic Web [27]. There are several reasons for this large scenario of applications. Ontologies provide a common terminology, over a domain, necessary for communication between people and organizations and also provide the basis for interoperability between systems. They can be used for making the content in information sources explicit and serve as an index to a repository of information [28]. The growing interest in ontologies, triggered the development of Ontological Engineering, a novel field concerned with the ontology development process, the ontology life cycle, the methods and methodologies for building ontologies, and the tool suites and languages that support them [29,30].

Despite the cited advantages, the choice of ontologies and formal representations incurs considerable costs for the retooling and upgrade of resources, and for the training of ontology developers. One serious problem is that differing ontologies may be developed and applied for the representation of the same domain. However, the mere use of ontology obviously does not warrant the elimination of heterogeneity; instead it can raise heterogeneity problems to a higher level. Ontology alignment, or ontology matching [31], a process that determines correspondences between concepts in different ontologies, can help to overcome those problems. In biology the heterogeneity of ontologies represents an emergent issue. In this respect, the OBO Foundry initiative [32] engages developers of science-based ontologies in the pursuit of a set of common principles for ontology development, with the goal of creating a suite of orthogonal interoperable reference ontologies in the biomedical domain.

The use of the word ontology within biology is relatively recent. Initially, computer scientists recognized in biological data a domain in which ontologies were needed in order to solve problems of heterogeneity. The second phase saw the adoption of bio-ontology by the biological community itself as a mean to consistently annotate different features, from genotype (e.g nucleotide sequences, proteins) to phenotype (e.g. diseases) [33]. Later, with the beginning of genome-scale sequencing projects and the diffusion of high-throughput experiments the amount of accessible biological data started to grow exponentially. Data are now dispersed throughout several different databases and their interpretation and analysis require sophisticated tools for data management and information processing. Organized in this way biological information is encapsulated within database schemes and is not easily available to scientist. Instead knowledge can be better captured and made available to both humans and computers thanks to ontologies Bio-ontologies are indeed fundamental components in biological data integration and annotation. In the last decade, several groups have been developing controlled vocabularies and descriptors mainly for the annotation of this kind of data. For instance, the Metabolomics Standards Initiative (MSI) ontology working group is developing an ontology to facilitate the consistent annotation of metabolomics experimental data [34]. Besides the well known Gene Ontology [24] there are many other initiatives focused on standardization and ontology development that may be cited, such as MIAME [35] and PRIDE [36]. These are mainly centred on the development of ontologies and bioinformatic tools for biological data annotation. However, only a few projects have been developed for the representation and formalization of the experimental protocols and the automatic operations producing such experimental data. A formal definition of scientific experimental design, laboratory entities and operations is undoubtedly important, also in the case of manually executed experiments. The development of an ontology of experiments is a fundamental step in the formalization of science, since experimentation is one of the most characteristic feature of science.

In this regard, the EXPO ontology of scientific experiment has been developed to formalize generic knowledge about scientific experimental design, methodology and representation of results [37]. The Ontology for Biomedical Investigations (OBI) addresses the need for controlled vocabularies not only for the experimental data annotation but also for the representation of investigations in the Biological and Biomedical Sciences [38]. Ontology represents the design of an investigation, the protocols and instrumentation used, the material used, the data generated and the type of analysis performed.

EXACT [39] is an ontology of experimental actions that can be used as a formalism suitable for a structured representation of laboratory protocols. The core of this structured vocabulary is a hierarchical classification of experimental actions based on goals of actions: the goal of separation, the goal of transformation and the goal of combination.

Exploiting the properties of EXACT in representing protocols, we expand and then combine its formalism with another, that of workflows to define a strategy that can be more expressive and efficient in protocol formalization.

### Workflows

In the workflow context, a process can be considered as the set of activities performed by different entities and their execution ordering through different constructors, which permit flow of execution control (e.g. sequence, choice, parallelism and join synchronization). An elementary activity is an atomic piece of work [40].

A workflow is therefore the structured definition of a process used for the automatic management of particular activities. The formalization of a process (workflow schema) involves the definition of activities, the specification of their order of execution (i.e. the routing or control flow) and of the responsible actors. Other features should be taken into account too, e.g. the data flow [40] or the various ways in which resources are represented and utilized in workflows [41].

There are three well established formalisms applied for the specification/modelling of processes: Business Process Execution Language (BPEL), Business Process Modelling Notation (BPMN), XML Process Definition Language (XPDL).

BPEL [42] is an execution language based on XML specification for the formal description of business processes based on Web Services.

BPMN [43] is a graphical notation based on intuitive flowcharts for the definition of business processes. Originated from the Business Process Management Initiative, in 2005 it was merged into OMG [44] and in 2007 the version 1.1 became a standard.

XPDL [45] is a markup language created to ensure interoperability among different workflow management tools in order to handle workflow processes. It was designed to permit the exchange of process definitions, addressing both the graphical and the semantic notations of the relevant workflow. Born as a support for serialization of BPMN constructs, it also incorporates also information relating to the graphical representation (e.g. the position of blocks in the workflow). XPDL was developed by the Workflow Management Coalition (WfMC) [46], a consortium formed to define standards for the interoperability of workflow management systems.

In the last few years the interest for workflow development has seen a considerable growth in the scientific community [47]. Scientific workflows can be considered as the executable description of scientific processes [48]. Similar in nature to business workflow, they have the distinct characteristic of operating on large amounts of heterogeneous data. In particular, they are generally data-flow oriented instead of being event-based, and very versatile in composing flows of execution. In bioinformatics, in particular, workflows are extremely valuable for programming the steps of *in silico *experiments in a visual intuitive manner. However workflows are still not commonly adopted in the formalization of protocols for biological laboratory experiments.

There are several available tools for workflow design and enactment [49], for instance JPEd [11], an open-source visual editor for general-purpose workflows. Taverna [50], developed by the myGrid project, is the workflow platform most commonly used for the systematic analysis of vast amounts of data, but it does not allow description of laboratory experimental procedures. Taverna workflows can be shared among the scientific community thanks to Web 2.0 initiatives like myExperiment [51]. This social web site enables scientists to publish their workflows and in addition to execute, reuse and share workflows of other groups. In this way myExperiment contributes in reducing time-to-experiment, in sharing knowledge and expertise and in avoiding reinvention [52].

### The proposed approach

We propose to combine ontologies and workflows for formalizing protocols used in biological laboratories. Workflows permit an intuitive representation of protocols, allowing the synchronization of different executors. Our workflow specifications can be stored and shared using the XPDL standard interchange language [45]. By means of ontologies, laboratory knowledge can be directly embedded into the workflow model and shared using the standard OWL model, the Ontology Web Language [53]. In this manner the precise constraints defined in the ontologies are transferred to the protocol building blocks.

To allow the integration of workflow and ontologies we have developed the COW tool, an add-on for the JPEd workflow editor that permits an easy and intuitive design of "ontologized" workflows. This allows a formal representation of laboratory protocols dealing with specific equipment and operations.

## Results

### Combining Ontologies and Workflows

Our starting point is the EXACT ontology. We advocate its intrinsic value for describing protocols in a precise and unambiguous way and compared to OBI, it seems to be a better choice. The former describes the most typical entities of biomedical investigations undertaken by humans and not directly relevant to protocols. The latter is designed to specifically define experimental actions that can be performed by both scientists and machines and therefore it is more suitable for automation. Relying on ontologies, laboratory domain knowledge can be effiectively shared among the scientific community including scientists, computers and robots. The value of clearly describing protocols is demonstrated by the ability to exchange and compare them [39]. Ontologies provide the human and machine-understandable universal language for such a shared understanding. Using the EXACT vocabulary, the protocols become unambiguous and key elements of actions are precisely identified. For instance in an EXACT *Move *action, "what is moved?", "from where?" and "to where?" can be precisely defined.

On the other hand, defining protocols only by means of formal ontologies presents important limitations. Fully formalized protocols usually span many pages of text and the production of such descriptions by hand results labour intensive, error-prone and uninspiring [39]. The modular structure of laboratory processes is not well identified, and this does not facilitate the reuse of well defined "building blocks". Furthermore, in our case the command actions currently defined by EXACT are minimal not allowing loops and other complex constructs.

It would be very problematic and almost impossible, for human users to read and fully understand protocols defined only in formal languages. A synthetic description in natural language of each activity should remain associated with its formal description. This would make each step understandable at first sight also to non-ontology experts. In general for human scientists it would be desirable to have a tool that permits a visual overview of the whole protocol. This would allow an easy identification of the constituting blocks, of the flow of execution and of the executors of each activity. In addition the tool would permit the retrieval of detailed information for each single block (i.e. parameters of an action or the structure of a subprotocol).

Therefore we developed a domain-specific language (DSL) [54], together with software supporting it, that allows laboratory protocols to be expressed more clearly than the pre-existing languages presently allow. Keeping the advantages of ontologies, we adopt a more expressive formalism able to describe different aspects of laboratory protocols (i.e. execution flow and error handling). Among the existing models we opted for workflows which combine higher expressivity at the flow control level with higher comprehensibility for human beings. The combination of ontologies and workflows gave us the possibility of defining laboratory protocols in terms of workflows enriched with ontological knowledge.

Among the various standards available in the workflow community, we adopted XPDL which is the standard defined to facilitate interoperability between business processes and to promote serialization of the graphic BPMN notation.

We chose the EXACT ontology because it defines precise semantics for laboratory activities, while workflows are mainly designed to orchestrate them. In the literature, there was no evidence about methods specifically developed for building workflows based on ontologies. Our idea consists in taking elements of the EXACT *Action *class as building blocks (i.e. activities) of workflows, and using the EXACT *Equipment *class as parameters of the actions. Actions are executed using XPDL Applications (ranging from a text editor to custom built applications) to execute the whole protocol.

For integrating these two formalisms we applied the principles of model driven engineering (MDE) [55]). In MDE there are three kinds of models: model, metamodel and metametamodel. "A metametamodel (also called M3) is a model that is its own reference model (i.e. it conforms to itself). A metamodel (M2) is a model such that its reference model is a metametamodel. A terminal model (M1) is a model such that its reference model is a metamodel" [56]. The real-world manifestation of a model is also called M0. Our idea was to relate the two metamodels, XPDL and EXACT, to establish semantic correspondences between respective elements.

**Figure 1:**
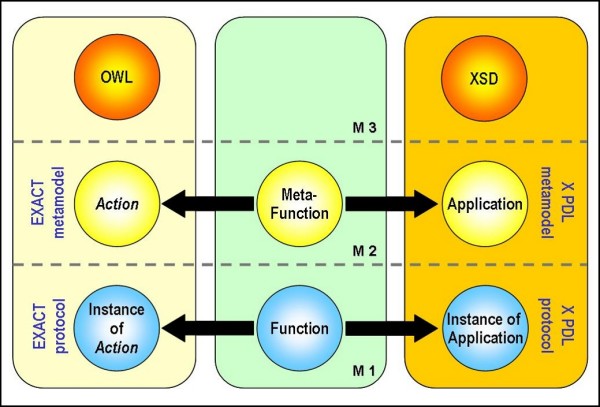
**Mapping between the two metamodels: EXACT Action and XPDL Application**.

XPDL can be considered as our first metamodel defined in XSD, which is its metametamodel (Figure 1). Therefore the XPDL language constructs, including Application and Activity, belong to the M2 level. In M1 we place Application definitions and invocations and Activity instances. An Activity represents an action which will be performed by a combination of resources and/or computer applications. One of the ways to implement an XPDL Activity is by using an XPDL Tool defined as a set of Applications. The latter are the description of programming language interfaces which may be invoked to support the Activity. The definition of Application reflects the interface that should be used to call the specific services that execute the Activity, including any parameters to be passed [57].

Our second metamodel is EXACT that has OWL as its metametamodel (Figure 1). In EXACT, the *Action *class contains concepts describing what an action can do on the basis of the goal of the actions. These concepts represent the effect of an action but do not give any information about how to obtain this effect. Therefore *Action *subclasses are abstractions of actions and do not represent the real-world actions.

Following this interpretation, the *Action *individuals belong to the M1 level. Since EXACT and XPDL are both placed at the M2 level, we applied a model-to-model transformation from the EXACT meta-model to the XPDL meta-model that permits their integration.

The concept of function in programming languages can help to understand the relationship between EXACT and XPDL. The function definition and invocation belong to the M1 level, whereas the grammar rules applied for writing the function are at the M2 level. In our case, instances of the *Action *class can be thought as functions, and the *Action *class as meta-function since it describes what a function is and does; for the same reason an Application is a meta-function and its instances are functions. Establishing a relationship between the EXACT *Action *class and XPDL Application allows the integration of the semantic of the ontology into workflows.

In EXACT each action, included in the *Action *class, has a list of *ad hoc *defined properties that specify which (and how) objects are to be manipulated in the action. In this way the formal parameters required for each *Action *class are specified at the class level, while the actual parameters are passed at the instance level. An example is the *Move *action, defined as "an experiment action to change a spatial location of an entity from a start location to an end location". For creating a *Move *instance we need to define the actual parameters: a start location, an end location and an object that is changing location. This formalization has a limitation: for each action reported in a laboratory protocol, we have to create a particular instance of the action specifying the actual parameters to be passed.

XPDL uses a well known standard mechanism for passing parameters based on IN, OUT and INOUT modes. Both formal and actual parameters are respectively specified and passed at the instance level. Starting from the above consideration EXACT can not be directly integrated into XPDL due to the different mode of parameters handling.

In order to permit the integration we have extended EXACT in two ways: adding a new ontology layer named UnGap and enriching the *Equipment *class with new subclasses. Starting from the *Action *class, we develop UnGap, an ontology layer that gives a new structure for parameter definition, so that ontology actions can correspond to XPDL Applications. The resulting mapping is represented in Figure 1. As a result each action is characterized by a list of input parameters (ParamIN), a list of output parameters (ParamOUT) and a list of parameters both of input and output (ParamIN_OUT).

In the new layer we also define the new class *Datatype *specifying the available types of parameters. This class contains the EXACT *Equipment *class and *owl:DatatypeProperty*. The former contains objects that can be manipulated by actions; the latter scalar values that can be requested from actions (e.g. *μ*l to be added). The instances of these subclasses can be used as actual parameters of XPDL Applications. The purpose of the UnGap layer is actually that of "closing the gap" between EXACT and XDPL. Thanks to the UnGap definition, EXACT and XPDL elements are now compatible. Therefore, action individuals correspond to XPDL Applications and action datatypes to XPDL datatypes. A relationship can be established between instances of *Action *and Application. In this way we can transform every instance of *Action *into the corresponding Application.

**Figure 2:**
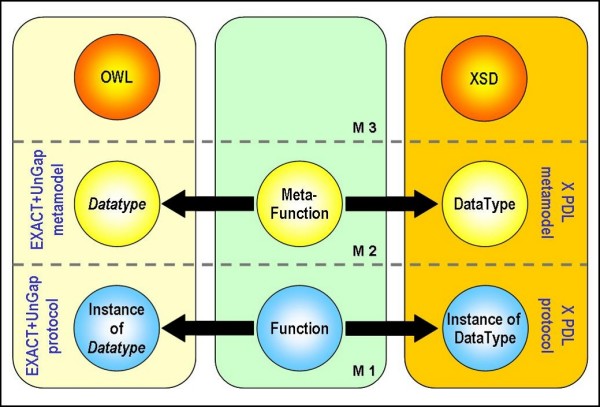
**Mapping between the two metamodels: UnGap Datatype and XPDL DataType**.

This translation mechanism provides a set of XPDL Application constructs that are invokable by workflow block activities. The invoked Application requires the complete specification of its formal parameters. This is obtained filling them with actual parameters defined as variables of subtype of the *Datatype *class. In this case we follow the same considerations for models and metamodels applied for the EXACT *Action*-XPDL Application mapping. We obtain a mapping between the UnGap *Datatype *class and XPDL DataType (Figure 2).

To exploit the advantages of ontologies in describing domain knowledge we decided to maintain *Datatype *instead of translating it into an XPDL construct. We linked each *Datatype *subclasses to new custom datatypes. In XPDL this can be conveniently implemented in the DataType using xpdl:ExternalReference construct.

**Figure 3:**
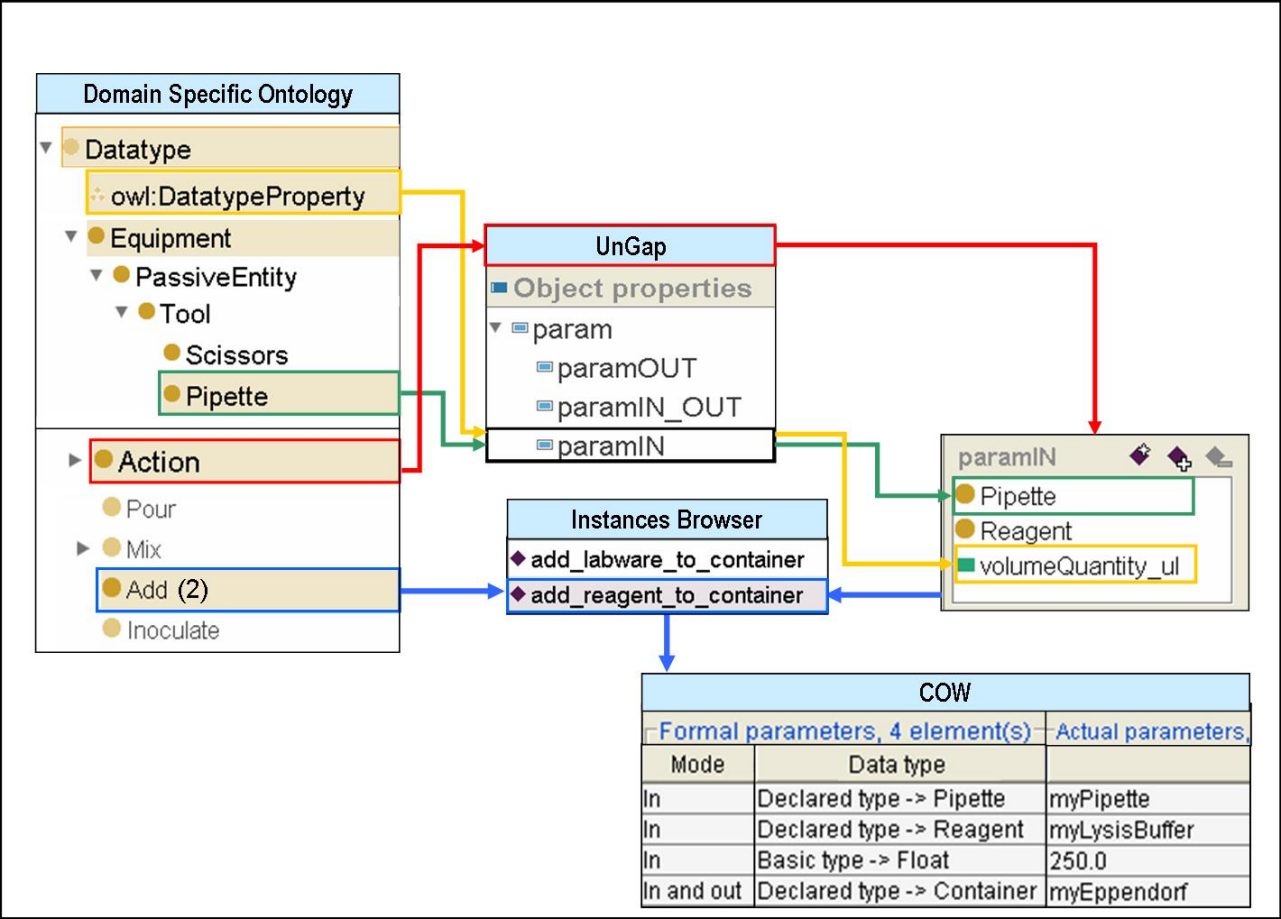
**Description of the system proposed for designing a formal laboratory protocol**. *Add *and add_reagent_to_container are respectively an *Action *subclass and one of its instances. *Pipette *is an *Equipment *subclass and is a formal parameter (paramIN) in add_reagent_to_container. myPipette is an OWL individual of *Pipette *class and it is also an actual parameter of the add_reagent_to_container XPDL Application.

Following this strategy, the UnGap layer permits us to reap the advantages of both ontologies and workflows and makes EXACT semantically and operationally integrable with XPDL. Now in order to execute an activity we can invoke a specific instance of *Action *(e.g. add_reagent_to_container) through the corresponding XPDL Application that has been translated with COW (Figure 3).

**Figure 4:**
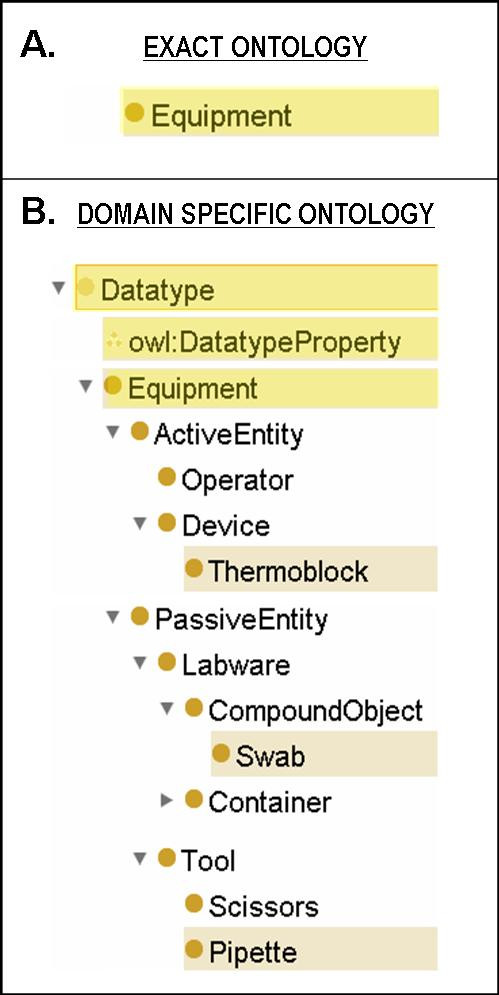
**The Equipment class**. A) In the EXACT ontology the *Equipment *class does not have any child term. B) In our Domain Specific Ontology the original *Equipment *class is, together with the owl:DatatypeProperty, a subclass of the new defined *Datatype *class. *Equipment *has been enriched with new defined concepts usually adopted in textual laboratory protocol (e.g. *Thermoblock*, *Swab *and *Pipette*).

Following the rules defined in UnGap, we can develop domain specific ontology (DSO) defining equipment and actions that are specific to a given laboratory. Figure 4 presents the modified DSO structure and its new included concepts: *ActiveEntity*, describing the entities able to perform actions and *PassiveEntity*, corresponding to objects of actions. As a result, we enrich the *Equipment *class with new concepts common in laboratory protocols like thermoblock and swab.

In this way we defined a mechanism for the automatic transformation between elements of our two base meta-models: EXACT *Action *corresponds to XPDL Application and individuals of *Action *correspond to instances of Application.

The protocols formalized with our method will be translated into workflows written in XPDL and their activities will be formalized by EXACT actions and saved in an OWL file.

### A COW protocol for paternity test

In order to discuss the novelty and the advantages of our proposal we present how the methodology and the developed tool have been used for formalising a laboratory protocol developed by our group.

**Figure 5:**
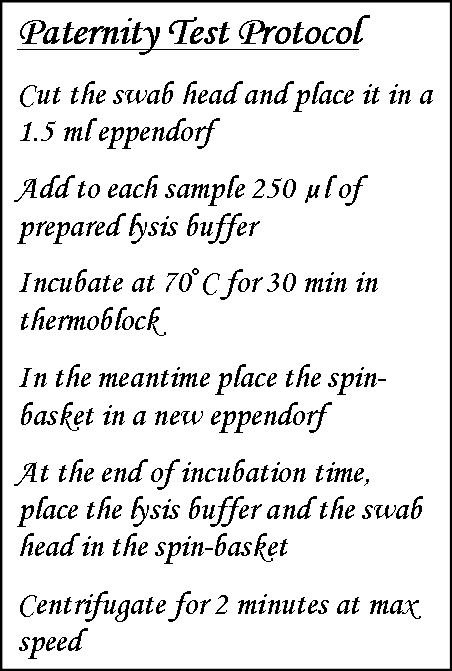
**The Paternity Test protocol (a fragment) in textual form**.

**Figure 6:**
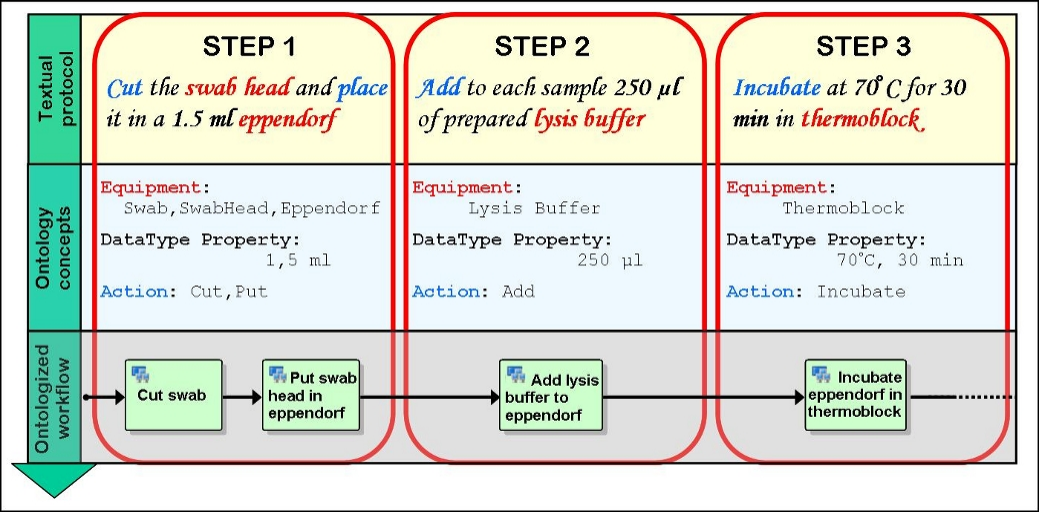
**The Paternity Test protocol represented using COW**. The first row represents the first three steps of the textual protocol. The second row represents the ontological concepts retrieved from the textual protocol. The third row displays an "ontologized" workflow where a single block corresponds to an action reported in the textual protocol. A single step (e.g. STEP 1) can be represented by more than one activity block.

In our laboratory we apply a protocol for paternity testing (Figure 5). The objective of the test is to confirm or exclude paternity relationship among the donors of two DNA samples. The protocol was set up and is followed by the wet-lab staff of our laboratory. It is composed of several detailed steps, required to perform a series of operations and involves various instruments to execute these operations. The first three steps of the protocol written in natural language are reported in the first row of Figure 6. These steps describe a series of actions which require specific preconditions and parameters for their execution.

In order to represent the protocol as a workflow, the first operation consists in accurately examining each sentence of the textual protocol which can be divided into numerous independent steps (first row of Figure 6). Reading step by step, we extract ontology concepts for *Action *and *Datatype *classes that should be included in our model (second row of Figure 6). In the textual form, the verbs (e.g. Cut, Add, Incubate) describe the nature of the action to be executed, the objects (e.g. Eppendorf, Thermoblock, SwabHead) represent the required equipment, and the parameters of actions (1.5 ml, 30 minutes) become *owl:DatatypeProperty*.

Each identified verb is a candidate for being included as an ontological action in our DSO. For any action not yet existing in the DSO, a new concept specific for that action is then inserted as an *Action *subclass. Following the EXACT rules, the newly defined *Action *subclasses can be classified, according to their nature, among the separation actions, the transformation actions or the combination actions. This solution permits us to define instances of *Action *subclasses. As defined in the UnGap ontology layer, for each instance of the *Action *class it is required to specify *ad-hoc *parameters among the types listed in the *Datatype class*.

In our ontology all the instances of the *Action *class share the properties of the class but can be distinguished by the specified parameters, as commonly applied in object-oriented programming (OOP). In Figure 3, the *Add *class has two instances: add_labware_to_container and add_reagent_to_container. The add_reagent_to_container instance corresponds to step 2 of our protocol and differs from the add_labware_to_container in the paramIN, paramOUT, paramIN_OUT parameters. The paramIN parameters include the *Pipette *and *Reagent *as *Equipment *classes and the *volumeQuantity_ul *as a *owl:DatatypeProperty*. It is worth underlining that the add_reagent_to_container is a "template" that is defined just once at the beginning of the formalization process. As a result it can be re-used to model every step in the protocol that requires the addition of a reagent to a container.

Using the COW tool, all the concepts and properties described in the DSO and UnGap layers can be used to design a workflow (third row of Figure 6). The resulting workflow is characterized by a flow of execution where activity blocks refer to the respective action in the corresponding protocol step. To specify a single block, e.g. the *Add *activity of step 2, we have to define workflow variables (myPipette, myLysisBuffer) that permit us to instantiate the add_reagent_to_container template. Such variables are instances of DSO classes, respectively *Pipette *and *Reagent *and they are actual parameters of the XPDL Application corresponding to the template. This method is repeated for each single protocol step obtaining a workflow completely defined by XPDL elements. The execution of the protocol formalized using COW can then be delegated to an independent XPDL-compliant run-time environment. The execution can also be concurrently distributed among several computational units interfaced with robotized stations or laboratory operators.

## Discussion

Laboratory automation is becoming increasingly crucial in many fields of experimental research. For instance, biological sample preparation often relies on robotic handling, while bioinformatics can greatly benefit from automatic procedures for analysis and management of data. We defined a method to construct workflows built on ontologies and designed it for the specific representation of laboratory protocols. The first part of our work results in the improvement of EXACT vocabulary and structure and in the creation of the UnGap layer. The latter permits us to apply a transformation from EXACT to XPDL and therefore to design an ontologized workflows. Later, for the implementation of our method we developed the COW tool (version 1.0) and validated it on the Paternity Test protocol used in our laboratory.

The formal definition of laboratory entities and operations, also in the case of manually executed experiments, presents undeniable advantages. Our method permits to obtain a structured representation of laboratory protocols, exploiting the advantages of both domain specific ontologies (DSO) and workflows. In particular, the adoption of a formal language defined in DSO helps to remove ambiguity and redundancies as well as contributing to exchange and comparison of laboratory knowledge. Moreover, by integrating the ontologies, the workflows permit us to define protocol execution flows by graphically designing the activities and their transitions. This approach allows the repeatability of experiments and enables the reliable interchange of experimental methods [37].

A critical point in the development of our method has been the choice of the formal language for use in the specification of experiments. The choice needs to strike a good balance between the desire for high expressivity and the need to avoid untenable computational complexity. We chose OWL-DL, which is especially designed to provide the maximum expressiveness possible while retaining computational decidability, we overcome calculability issues. Moreover, our ontology has a limited number of classes, which also have modest computability needs. There are several factors that complicate also the definition and the application of a domain specific language (DSL). It initially requires domain and language development expertise and only very few people have both. Other limitations include the development of training material, language support and maintenance [58]. Another limitation of our approach is the formalization of the legacy protocols that can be labour intensive and require the collaboration of both wet-lab and computer scientists. These aspects lead to challenging and time-consuming stages of the project development.

At the time of this writing the EXACT-XPDL mapping is not yet complete because not all the EXACT constructs have been mapped to the XPDL corresponding ones. Consequently, the currently mapped constructs are insufficient to formalise and represent a laboratory protocol in all its aspects. Moreover the DSO lacks additional concepts necessary for complete coverage of the entire laboratory domain. For example, our ontology includes concepts about actions and equipment, but does not yet represent concepts like pre- and post- conditions of actions. Further work is therefore needed to complete both the mapping and the ontology to finally allow for a comprehensive representation of laboratory protocols.

The effort required by a scientist to represent an experiment using our proposed platform ultimately depends on the available level of formalized laboratory knowledge. Undoubtedly the set-up of the platform requires ontology building skills and precise knowledge of the domain. Our system has been tested by some wet-lab scientists who provided suggestions and feedback for possible improvements. At this stage, the user needs preliminary training from COW developers. The trainers should provide support for designing the protocol, mostly by ensuring that all required parameters, dependences and constrains are captured and set. However, once the platform has been tested by a sufficient number of wet-lab users and improved based on their feedback, the procedure for the modelling of experiment protocols will be considerably simplified and external support will no longer be needed. Using COW, the protocol definition may be done directly and easily by wet-lab scientists who are typically non-programmers - though this need not be the case - but are instead more conversant with laboratory domain knowledge. They may write protocols without writing code, which keeps the resulting specification clear and easy to read and makes maintenance much easier. The final objective of our project is actually the development of an integrated environment for the design, analysis, tracing and simulation/execution of laboratory protocols. At run time, the execution platform would eventually orchestrate software and robotized stations. We envision the existence of a compiler able to translate protocols formalised using the COW tool into executable systems. The definition of such a compiler is still in progress and for this reason, the automation of experiments has not yet been tested. In our system we also plan to introduce dynamic management of resources during the execution of experiments, considering the availability and the workload of multiple executors that are able to perform a scheduled activity. To foster protocol interoperability among laboratories, it is essential to avoid dependency on execution environments. During design therefore, the user should provide the protocol structure as a set of actions without specifying any environment-dependent parameters such as the exact kind of machine that can be used.

## Conclusion

Using COW, a laboratory protocol may be directly defined by wet-lab scientists without writing code, which will keep the resulting protocol's specifications clear and easy to read and maintain. Further work is required to finally allow a comprehensive representation of laboratory protocols and therefore we are currently working to improve the COW tool with the implementation of static code analysis functionalities and with a new graphical user interface. At the same time we are working on a compiler able to translate a protocol defined by means of COW tool in an executable version that takes in account also platform specific details.

## Methods

### The COW software

**Figure 7:**
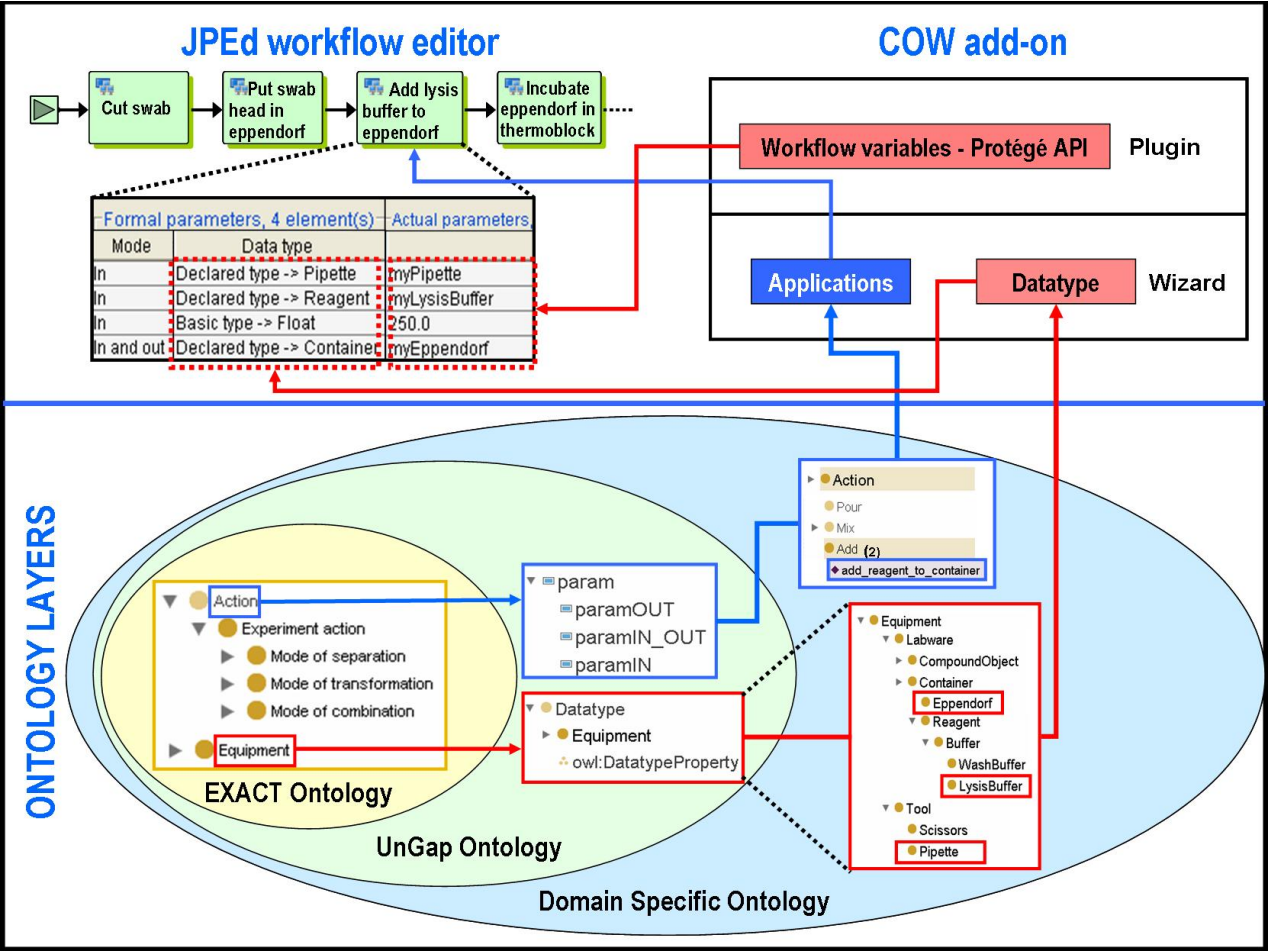
**The COW tool**. Architecture of the system proposed for designing formal laboratory protocols. The COW add-on interfaces the JPEd workflow editor with laboratory ontologies. *Add *and add_reagent_to_container represent respectively an example of a domain specific *Action *subclass and one of its instances.

COW comes in the form of a wizard and a plugin and uses ontologies modelled in OWL. Our software has been developed in Java (version 1.5). Protégé (version 3.4) has been used for the modification of EXACT and the creation of UnGap layer and DSO. The COW tool is distributed as an add-on for JPEd with minor changes provided by us (version: 2.0.1-SNAPSHOT). In the wizard, ontology concepts are transformed into XPDL version 1.0 (Figure 7).

The EXACT ontology represents the kernel of laboratory formalized knowledge. On top of this a second ontological layer named UnGap has been added. It extends the concept of *Action *enriching it with a precise schema for uniformly managing the generic list of parameters for each possible action (i.e. paramOUT, paramIN_OUT, paramIN). A user can develop his own DSO over UnGap. In DSO, in particular, the *Action *class can be specialized according to the specific nature and needs of the laboratory. The wizard component of the add-on translates each subclass of *Datatype *into a XPDL DataType.

Furthermore, each individual of the *Action *ontology class is transformed into XPDL as an *Application *construct.

Workflows in XPDL can contain variables of the standard types (e.g. integer, strings) but also of any user defined type. Therefore, as the wizard automatically converts a subclass of *Datatype *into XPDL DataType, the plugin allows the declaration of workflow variables of a type coming from the ontology. Users can create such types of variable also on the fly, thanks to the Protégé API [59]. They enable the use of the Protégé GUI modules for dealing with the OWL model and populating the DSO with individuals, simultaneously with the workflow development.

When beginning a new protocol definition, the user is required to provide the location of the DSO to be used. An ontology *Action*, mapped into a XPDL *Application*, can be retrieved in the JPEd Application Panel. Choosing a specific application from the shown list, it is possible to see a general description of the application, including its parameter list with related type. An application can finally be invoked from an Activity block of a workflow process. COW enables us to save the protocols as "ontologized" workflows, creating an OWL file for ontology individuals as well as the usual XPDL file.

## Competing interests

The authors declare that they have no competing interests.

## Authors' contributions

NC, GV, BS conceived the research project. AM and MR identified the technical solutions for combining ontologies and workflows as well as designing the architecture of the COW tool. AM and MR developed the UnGap layer and COW under the supervision of NC and TV. EF worked on the domain specific ontology and on the case study. EF, AM, MR, NC drafted the manuscript. All authors critically revised and approved the final document.

## References

[B1] Joshua L Alexa M "Ome Sweet "Omics-A Genealogical Treasury of Words. -- AccessMyLibrary -Promoting library advocacy The Scientist 2001

[B2] Tyers M Mann M From genomics to proteomics Nature 2003 422 193 197 10.1038/nature01510 10.1038/nature01510 12634792

[B3] Baldi P Hatfield GW Hatfield WG DNA Microarrays and Gene Expression: From Experiments to Data Analysis and Modeling 2002 1 Cambridge University Press

[B4] Fields S PROTEOMICS: Proteomics in Genomeland Science 2001 291 1221 1224 10.1126/science.291.5507.1221 10.1126/science.291.5507.1221 11233445

[B5] Goodacre R Vaidyanathan S Dunn WB Harrigan GG Kell DB Metabolomics by numbers: acquiring and understanding global metabolite data Trends Biotechnol 2004 22 245 252 10.1016/j.tibtech.2004.03.007 10.1016/j.tibtech.2004.03.007 15109811

[B6] Shulaev V Metabolomics technology and bioinformatics Briefings in Bioinformatics 2006 7 128 10.1093/bib/bbl012 10.1093/bib/bbl012 16772266

[B7] Muggleton S 2020 Computing: Exceeding human limits Nature 2006 440 409 410 10.1038/440409a 10.1038/440409a 16554781

[B8] Steering the future of computing Nature 2006 440 7083 383 10.1038/440383a 16554760

[B9] De Roure D Hendler JA E-Science: the grid and the Semantic Web Intelligent Systems, IEEE 2005 19 65 71 10.1109/MIS.2004.1265888

[B10] King RD Rowland J Oliver SG Young M Aubrey W Byrne E Liakata M Markham M Pir P Soldatova LN Sparkes A Whelan KE Clare A The Automation of Science Science 2009 324 85 89 10.1126/science.1165620 10.1126/science.1165620 19342587

[B11] JaWE based process editor http://www.jped.org

[B12] Science Advisory Board http://www.scienceboard.net/

[B13] Protocol-online http://www.protocol-online.org

[B14] Nature Methods http://www.nature.com/nmeth/index.html

[B15] Journal of Visualized Experiments http://www.jove.com

[B16] Kritikou E Watch and learn Nat Rev Mol Cell Biol 2007 8 4 http://www.nature.com/nrm/journal/v8/n1/full/nrm2097.html 10.1038/nrm2097 10.1038/nrm2097

[B17] Nature Procotols http://www.nature.com/nprot/index.html

[B18] Huang DW Sherman BT Lempicki RA Systematic and integrative analysis of large gene lists using DAVID bioinformatics resources Nature protocols 2008 4 44 57 10.1038/nprot.2008.211 10.1038/nprot.2008.211 19131956

[B19] Fiegler H Redon R Carter N Construction and use of spotted large-insert clone DNA microarrays for the detection of genomic copy number changes Nat Protocols 2007 2 577 587 10.1038/nprot.2007.53 10.1038/nprot.2007.53 PMC2688820 17406619

[B20] Liu L Özsu MT Encyclopedia of database systems 2009

[B21] Malucelli A Palzera D Oliveiraa E Ontology-based Services to help solving the heterogeneity problem in e-commerce negotiations Electronic Commerce Research and Applications 2006 5 29 10.1016/j.elerap.2005.08.002 10.1016/j.elerap.2005.08.002

[B22] Hecker M Dillon T Chang E Privacy Ontology Support for E-Commerce IEEE Internet Computing 2008 12 54 61 10.1109/MIC.2008.41 10.1109/MIC.2008.41

[B23] Hepp M De Leenheer P De Moor A Ontology Management: Semantic Web, Semantic Web Services, and Business Applications (Semantic Web and Beyond) 2007 7 1 Springer

[B24] Gene Ontology http://www.geneontology.org/

[B25] Bard J Rhee S Ontologies in biology: design, applications and future challenges Nature reviews Genetics 2004 5 213 222 10.1038/nrg1295 10.1038/nrg1295 14970823

[B26] Brewster C O'Hara K Fuller S Wilks Y Franconi E Musen M Ellman J Shum S Knowledge Representation with Ontologies: The Present and Future IEEE Intelligent Systems 2004 19 72 81 10.1109/MIS.2004.1265889 10.1109/MIS.2004.1265889

[B27] Arndt R Troncy R Staab S Hardman L Vacura M COMM: Designing a Well-Founded Multimedia Ontology for the Web ISWC/ASWC 2007 30 43 10.1007/978-3-540-76298-0_3

[B28] Lambrix P Habbouche M Pérez M Evaluation of ontology development tools for bioinformatics Bioinformatics 2003 19 1564 10.1093/bioinformatics/btg194 10.1093/bioinformatics/btg194 12912838

[B29] Perez AG Corcho O Lopez MF Ontological Engineering: with examples from the areas of Knowledge Management, e-Commerce and the Semantic Web. (Advanced Information and Knowledge Processing) 2004 First Springer

[B30] de Nicola A Missikoff M Navigli R A software engineering approach to ontology building Information Systems 2009 34 258 10.1016/j.is.2008.07.002 10.1016/j.is.2008.07.002

[B31] Euzenat J Shvaiko P Ontology Matching 2007 1 Springer

[B32] Smith B Ashburner M Rosse C Bard J Bug W Ceusters W Goldberg LJ Eilbeck K Ireland A Mungall CJ OBI Consortium Leontis N Rocca-Serra P Ruttenberg A Sansone SAA Scheuermann RH Shah N Whetzel PL Lewis S The OBO Foundry: coordinated evolution of ontologies to support biomedical data integration Nature biotechnology 2007 25 11 1251 1255 10.1038/nbt1346 17989687 PMC2814061

[B33] Bodenreider O Stevens R Bio-ontologies: current trends and future directions Briefings in bioinformatics 2006 7 256 274 10.1093/bib/bbl027 10.1093/bib/bbl027 16899495 PMC1847325

[B34] Metabolomics Standards Initiative http://msi-ontology.sourceforge.net/

[B35] Minimum Information About a Microarray Experiment http://www.mged.org/Workgroups/MIAME/miame.html

[B36] PRoteomics IDEntifications database http://www.ebi.ac.uk/pride/

[B37] Soldatova L King R An ontology of scientific experiments Journal of The Royal Society Interface 2006 3 795 10.1098/rsif.2006.0134 10.1098/rsif.2006.0134 PMC1885356 17015305

[B38] Courtot M Bug W Gibson F Lister A Malone J Schober D Brinkman R Ruttenberg A The OWL of Biomedical Investigations Proceedings of the Fifth OWLED Workshop on OWL: Experiences, 2008 2008

[B39] Soldatova L Aubrey W King R Clare A The EXACT description of biomedical protocols Bioinformatics (Oxford, England) 2008 24 i295 303 10.1093/bioinformatics/btn156 10.1093/bioinformatics/btn156 18586727 PMC2718634

[B40] Russell N Hofstede AH Edmond D der Aalst WM Workflow Data Patterns: Identification, Representation and Tool Support, Berlin/Heidelberg: Springer-Verlag, Volume 3716 2005 chap 2005 Chapter 23 Springer-Verlag 353 368

[B41] Russell N Aalst WMP van der ter Hofstede AHM Edmond D Workflow Resource Patterns: Identification, Representation and Tool Support, Heidelberg 2005 Springer Berlin 216 232

[B42] Business Process Execution Language http://www.bpelsource.com/

[B43] BPMI http://www.bpmi.org

[B44] The Object Management Group http://www.omg.org

[B45] XML Process Definition Language http://www.wfmc.org/xpdl.html

[B46] Workflow Management Coalition http://www.wfmc.org/

[B47] Deelman E Gannon D Shields M Taylor I Workflows and e-Science: An overview of workflow system features and capabilities Future Generation Computer Systems 2009 25 528 10.1016/j.future.2008.06.012 10.1016/j.future.2008.06.012

[B48] Shawn CB Bowers S Jones MB Ludäscher B Schildhauer M Tao J Incorporating Semantics in Scientific Workflow Authoring In Proceedings of the 17th International Conference on Scientific and Statistical Database Management (SSDBM'05) 2005

[B49] Romano P Automation of in-silico data analysis processes through workflow management systems Brief Bioinform 2008 9 57 68 10.1093/bib/bbm056 18056132

[B50] Oinn T Addis M Ferris J Marvin D Senger M Greenwood M Carver T Glover K Pocock M Wipat A Li P Taverna: a tool for the composition and enactment of bioinformatics workflows Bioinformatics (Oxford, England) 2004 20 3045 3054 10.1093/bioinformatics/bth361 10.1093/bioinformatics/bth361 15201187

[B51] myExperiment http://www.myexperiment.org/

[B52] de Roure D Goble C Software Design for Empowering Scientists IEEE Software 2009 26 88 95 10.1109/MS.2009.22 10.1109/MS.2009.22

[B53] Ontology Web Language http://www.w3.org/TR/owl-ref/

[B54] van Deursen A Klint P Visser J Domain-specific languages: an annotated bibliography SIGPLAN Not 2000 35 6 26 36 10.1145/352029.352035

[B55] France R Rumpe B Model-driven Development of Complex Software: A Research Roadmap FOSE '07: 2007 Future of Software Engineering 2007 Washington, DC, USA: IEEE Computer Society 37 54 full_text

[B56] Jouault F Bezivin J KM3: a DSL for Metamodel Specification Lecture Notes In Computer Science 2006 4037 171 185 full_text

[B57] Shapiro R Marin M Workflow Management Coalition Workflow StandardProcess Definition Interface- XML Process Definition Language. The Workflow Management Coalition, 99 Derby Street, Suite 200 Hingham, MA 02043 USA 2008

[B58] Mernik M Heering J Sloane AM When and how to develop domain-specific languages ACM Comput Surv 2005 37 4 316 344 10.1145/1118890.1118892

[B59] Protege Application Programming Interface http://protege.stanford.edu/plugins/owl/api/

